# Evaluation of the myocardial deformation in the diagnosis of rejection after heart transplantation

**DOI:** 10.3389/fcvm.2022.991016

**Published:** 2022-10-13

**Authors:** Rodrigo Cordovil Pinto Lobo da Costa, Ana Clara Tude Rodrigues, Marcelo Luiz Campos Vieira, Claudio Henrique Fischer, Claudia Gianini Monaco, Edgar Bezerra Lira Filho, Fernando Bacal, Adriano Caixeta, Samira Saady Morhy

**Affiliations:** Hospital Israelita Albert Einstein, São Paulo, Brazil

**Keywords:** heart transplantation, echocardiography, heart failure, ventricular dysfunction, graft rejection

## Abstract

**Introduction:**

Heart transplantation represents main therapy for end-stage heart failure. However, survival after transplantation is limited by development of graft rejection. Endomyocardial biopsy, an invasive and expensive procedure, is gold standard technique for diagnosis of rejection. Most of biopsy complications are observed using echocardiography. Novel echocardiographic techniques, such as myocardial strain and three-dimensional reconstruction, can be useful in heart transplant patients.

**Purpose:**

To evaluate ventricular strain in heart transplant patients and association with rejection, cellular or humoral, as well as two- and three-dimensional echocardiographic parameters.

**Methods:**

Cohort of patients from heart transplant program taken to echocardiography after endomyocardial biopsy, from December 2017 to January 2020. Ventricular strain and three-dimensional left ventricle parameters were studied. Rejection results were retrieved from medical record. Qualitative variables were expressed by absolute frequency and percentages, while continuous variables by means and standard deviations. Association between rejection and variables of interest was measured by odds ratio and confidence interval of 95%, with *p*-value < 0.05.

**Results:**

123 post-endomyocardial biopsy echocardiographic exams were performed in 54 patients. Eighteen exams were excluded, lasting 105 exams to be evaluated for conventional and advanced echocardiographic parameters. Male patients were 60.4%. Prevalence of cellular rejection was 8.6%, humoral rejection 12.4%, and rejection of any type 20%. There was no association between right ventricular strain and rejection, whether cellular (*p* = 0.118 and *p* = 0.227 for septum and free wall, respectively), humoral (*p* = 0.845 and *p* = 0.283, respectively), or of any type (0.504 and 0.446). There was no correlation between rejection and left ventricle global longitudinal strain, three-dimensional ejection fraction or desynchrony index. Conventional parameters associated to rejection were left ventricle posterior wall thickness [OR 1.660 (1.163; 2.370), *p* = 0.005] and left ventricle mass index [OR 1.027 (1.011; 1.139), *p* = 0.001]. Left ventricle posterior wall thickness remained significant after analysis of cellular and humoral rejection separately [OR 1.825 (1.097; 3.036), *p* = 0.021 and OR 1.650 (1.028; 2.648), *p* = 0.038, respectively].

**Conclusions:**

There was no association between ventricular strain, three-dimensional left ventricular ejection fraction and the desynchrony index and rejection, cellular or humoral. Evidence of association of graft rejection with left ventricle posterior wall thickness and left ventricle mass index was observed.

## Introduction

Heart transplantation (HT) represents the main therapy for end-stage heart failure, resulting in a survival rate of more than 90% one year after surgery (1). However, success of HT is limited by the development of acute/chronic graft rejection (GR) and cardiac graft vasculopathy, which represent the main causes of morbidity and mortality in these patients (2). Since GR is usually asymptomatic and has a rapid onset, regular surveillance of transplant patients is mandatory, particularly in the first year after HT. Currently, the reference test for the diagnosis of GR is endomyocardial biopsy (EMB), however, it is associated with significant complications (3). The incidence of graft rejection is around 20–40% and responsible for ~12% of deaths (4). Caused by a determined alloimmune process of the recipient against the histocompatibility complex antigens of the donor, acute GR can be cellular or humoral (antibody-mediated) (5). Acute humoral rejection occurs mainly at the end of the first post-transplant year and demands an aggressive therapy (6, 7) since it is associated with a worse prognosis (8).

Echocardiography is currently the most useful non-invasive imaging modality for the evaluation and monitoring of transplant patients, as it is widely available, low-cost, safe and well tolerated. The recent development of novel echocardiographic techniques has increased the probability of early detection of graft dysfunction (1). In addition, it provides accurate information on the anatomy and functioning of the graft (2). Despite being a strong predictor of events in heart transplant patients, LV ejection fraction (LVEF) is not an early indicator of graft dysfunction (9). Alternatively, the right ventricle (RV), plays an important role in the evaluation of heart transplant patients, with RV failure being the most important cause of death in the early days after HT (10).

Other echocardiographic techniques such as speckle tracking, derived from the two-dimensional image, and potentially more accurate after three-dimensional reconstruction, enable assessment of myocardial deformation (strain) (11). Several studies have assessed the use of strain on HT patients with noteworthy findings; while a reduced LV global longitudinal strain (GLS) observed immediately after surgery is associated with a higher cardiovascular risk (12), different clinical conditions can also lead to reduced GLS values even with normal LVEF (13–15). A gradual improvement on RV strain has been associated to a better graft function (16), whereas the combined deformation analysis of both ventricles and their correlation with rejection showed promising results (17). Recently, an association has been found between the reduction in ventricular strain and the presence of cellular rejection (18, 19). Other advanced echocardiographic tools, such as the three-dimensional (3D) LVEF and 3D LV dissynchrony index (ID3D) could add further value to the understanding of myocardial function in HT patients (20, 21). However, their role in rejection development and follow-up stays unclear. Therefore, we aimed to evaluate the association of RV and LV strain, along with 2D and 3D echocardiographic measurements with the presence of graft rejection.

## Materials and methods

### Population and study design

A prospective cohort study was carried out with patients from the Cardiac Transplantation Program at Hospital Israelita Albert Einstein (HIAE) who underwent transplant surgery at the same institution. All transplant patients admitted in Cath Lab for EMB from December 2017 to January 2020 were selected. Adult HT patients (over 18 years old) who underwent transthoracic echocardiogram (TTE) within 6 h after EMB were included in the study. Individuals who had poor image quality were excluded.

According to the HIAE protocol, HT patients underwent routine EMB, the first one usually performed on the seventh postoperative day (PO), when it is expected that the serum level of cyclosporine (calcineurin inhibitor, which inhibits the expression of interleukin-2 receptors, and limits lymphocyte proliferation and differentiation) reaches the reference value (350–450 ng/ mL). Then, EMBs are programmed to be held on the 14th PO and right before hospital discharge. Subsequently, EMBs are performed at day 45 of follow-up, 3, 6, and 12 months after transplantation. In addition to that schedule, other EMBs can be indicated depending on clinical manifestations, presence of infection or positive viral panel, each case individually evaluated. Each EMB was followed, within 6 h, by a TTE, as routine screening for procedure complications.

### Ethical aspects

The present study was carried out in accordance with the principles established by the Declaration of Helsinki, and its project approved by the Research Ethics Committee of HIAE. All patients had Informed Consent Term comprehensively read and signed, if agreed to participate. A copy of the term was attached to the patient's medical record. The research was conducted without cost or financial support to researchers or patients.

### Echocardiographic analysis

All TTE were performed on an EPIQ 7 (Philips, Koninklijke, Netherlands) by one of the HIAE echocardiographers and recorded on optical media for further offline analysis of morphological and functional echocardiographic parameters by a single experienced echocardiographer (RCPLC), on an institutional workstation through the TOMTEC RV 4D software (TomTec Imaging Systems, Unterschleißheim, Germany). Another EPIQ CVx device (Philips, Koninklijke, Netherlands) was also used for offline calculation of LV automatic GLS (auto-strain) and LV three-dimensional measurements (3D ejection fraction and desynchrony index). All professionals involved in the acquisition of images, preparation of the echocardiographic report and offline analysis were unaware of the clinical characteristics of the patients, as well as the histopathological results.

#### Conventional parameters

All two-dimensional echocardiographic measurements were obtained accordingly international validated guidelines (22). Except for technical limitations, exams were performed by obtaining images of the heart by usual echocardiographic views, resulting linear measurements of aortic root, left atrium (LA), RV and LV diameters, septal and posterior wall thickness. The assessment of valvular flows was performed using pulsed-wave, continuous-wave and color Doppler. It was also included identification pericardial effusion and pulmonary hypertension, if present. Quantification of biventricular systolic function was made by traditional methods.

#### Strain and morphological and functional analysis of the right ventricle

The assessment of right ventricular strain was performed using a 3D full-volume acquired with frame rate > 40 cycles-per-second and four-beat recording. Using the offline analysis software, the longitudinal alignment of the heart chambers in four- and two- chamber views was obtained, LV outflow tract diameter was measured, such as the distance between the mitral valve and the left ventricular apex and the distance between the tricuspid valve and the right ventricular apex, in addition to the RV basal diameter in short-axis acquisition and marking of the anterior and posterior cardinal points of the RV. After automatic detection of endocardial speckles and manual adjustments if needed, peak systolic strain of the RV free wall and septal wall were obtained. 3D volumetric and functional analysis of RV were also achieved, as well as RV dimensions, TAPSE and FAC.

#### Strain and morphological and functional analysis of the left ventricle

GLS was acquired through the analysis of its myocardial deformation obtained by four- two- and three-chamber views, after semi-automatic recognition of the endocardial borders and synchronization of the cardiac cycle.

The 3D evaluation of the LV, similarly to the RV, was performed with a full volume acquisition, four-beat optimized, at least 40 frames-per-second, with the patient in expiratory apnea. After LV landing marks in systole and diastole were indicated, LVEF-3D was automatically obtained, as well as the 3D desynchrony index.

### Endomyocardial biopsies

EMBs were performed at the HIAE Cath Lab by an experienced interventional cardiologist. After venous femoral access, under radioscopic guidance, from 5 to 10 RV endomyocardial fragments were collected for subsequent histopathological analysis, performed by an experienced pathologist without knowledge of the echocardiographic results. Histological specimens were graded from 0 to 3R for cellular rejection according to international guidelines (23) and considered positive if 2R. For the evaluation of antibody-mediated rejection, the histological fragments were submitted to immunohistochemistry and immunofluorescence analysis and were considered positive when reactive at any intensity to one of those methods.

### Statistical analysis

The results extracted from the database were evaluated, at first, regarding the presence or absence of rejection, whether cellular or antibody-mediated (humoral). Subsequently, the study was carried out by evaluating data segmented per type of rejection. Qualitative variables were described by number and percentages and quantitative variables were described by mean ± standard deviation, once the properties referring to the assumption of normality of the data were satisfied. The investigation of the variables of interest in the exams in relation to cellular rejection, humoral rejection by immunofluorescence or immunohistochemistry and rejection of any type was carried out using models of equations of generalized estimation with binomial distribution and interchangeable correlation structure, seeking to contemplate the dependence between the measurements of different exams of the same individual. The results were presented by odds ratio (OR), confidence intervals of 95% (95% CI) and *p*-values. Intra and interobserver variability was performed using the intraclass correlation index in a sample of 10 cases and considered excellent if >0.70. The analyzes were performed using the STATA/SE 15.1 software (StataCorp, 2017. College Station, TX- StataCorp LLC) and the significance level adopted was 5%.

## Results

**Figure 1:**
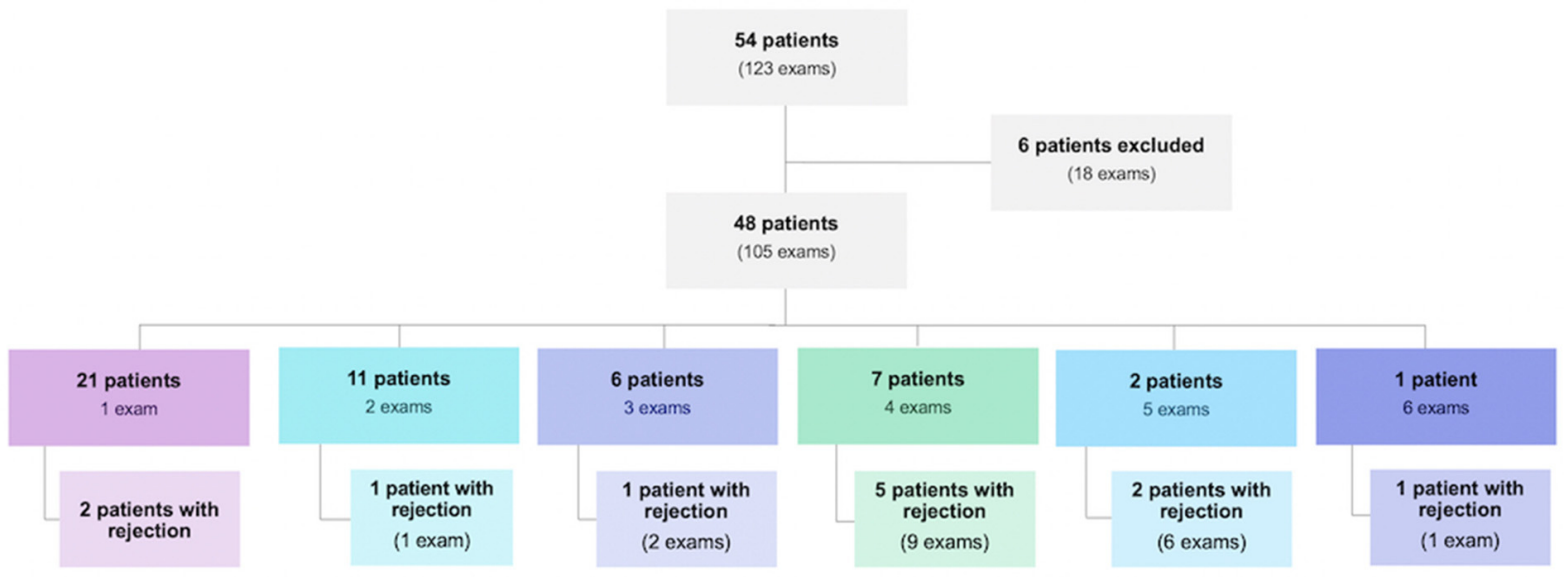
Population of heart transplant patients evaluated during the study period and their distribution regarding the number of endomyocardial biopsies performed and the presence of rejection.

From December 2017 to January 2020, 123 post-EMB echocardiographic examinations were performed in 54 HT patients at the institution. Of these tests performed, 18 were excluded because they had poor quality images. The 105 remaining exams corresponded to 48 patients, 29 were men (60.4%) and 19 were women (39.6%). The number of exams per patient ranged from one to six, as follows: one exam in 21 patients (43.8%), two exams in 11 patients (22.9%), three exams in 6 patients (12.5%), four exams in 7 patients (14.6%), five exams in 2 patients (4.2%) and six exams in one patient (2.1%) (Figure 1).

The overall prevalence of rejection was 20% (95% CI: 12.2%; 27.8%) in the population studied. The demographic characteristics of the patients were described in relation to the moment of the first examination. The mean age at the time of the examination was 50 years, ranging from 18 to 72 years. The weight varied between 43 and 93 Kg, with an approximate average of 68 Kg. The mean body surface area (BSA) was 1.77 m^2^ and the mean body mass index (BMI) was 24.1 Kg/m^2^ ranging from 17.7 to 34.2 Kg/m^2^ (Table 1).

**Table 1:** Clinical and demographic characteristics of the 48 patients included in the study.

**Demographic characteristics**	
**Gender**, ***n*** **(%)**	
Male	29 (60.4)
**Age years**	
Mean ± SD	50.3 ± 10.9
Minimum–maximum	18–72
**Weight (Kg)**	
Mean ± SD	67.6 ± 12.0
Minimum–maximum	43–93
**BSA (m** ^**2**^ **)**	
Mean ± SD	1.77 ± 0.18
Minimum–maximum	1.37–2.13
**BMI (Kg/m** ^**2**^ **)**	
Mean ± SD	24.1 ± 3.9
Minimum–maximum	17.7–34.2

ASC, body surface area; BMI, body mass index.

According to Table 2, it is observed that the measurement of the thickness of the left ventricular posterior wall (LVPW) [OR 1.660 (1.163; 2.370), *p* = 0.005] and the left ventricular mass index (LVMI) [OR 1.027 (1.011; 1.139), *p* = 0.001] significantly differentiated in the exams with rejection from those without. The other parameters did not show significant differences. Thus, it can be said that when LVPW increases 1 mm, the chance of rejection increases 66% and when LVMI increases 1 g/m^2^, the chance of rejection increases 2.7%.

**Table 2:** Conventional echocardiographic parameters obtained by two-dimensional and descriptive evaluation according to rejection.

**Echocardiographic parameter**	**General (*n* = 105)**	**Rejection**		**OR (95% CI)**	***p*-value**
		**Present (*n* = 21)**	**Absent (*n* = 84)**		
LVDD (mm)	45.5 ± 3.9	46.0 ± 4.7	45.3 ± 3.7	1.091 (0.968; 1.229)	0.152
LVSD (mm)	28.6 ± 3.0	28.9 ± 2.9	28.5 ± 3.0	1.002 (0.862; 1.165)	0.975
SIV (mm)	10.7 ± 1.6	11.5 ± 1.0	10.5 ± 1.6	1.286 (0.992; 1.669)	0.058
LVPW (mm)	10.2 ± 1.2	11.0 ± 1.0	10.0 ± 1.1	1.660 (1.163; 2.370)	0.005
LVMI (g/m^2^)	96.2 ± 19.8	107.3 ± 21.5	93.5 ± 18.5	1.027 (1.011; 1.139)	0.001
LVEF (%)	66.9 ± 5.4	67.3 ± 4.0	66.8 ± 5.7	1.048 (0.973; 1.128)	0.214
RVDD (mm)	27.5 ± 3.3	28.2 ± 2.7	27.3 ± 3.5	1.041 (0.920; 1.179)	0.522
PH, *n* (%)					0.056
Absent	86 (81.9)	13 (61.9)	73 (86.9)	1,000	
Present (>35 mmHg)	19 (18.1)	8 (38.1)	11 (13.1)	2.305 (0.978; 5.434)	
Pericardial effusion, *n* (%)					0.231
Absent	73 (69.5)	12 (57.1)	61 (72.6)	1,000	
Minimal/mild	30 (28.6)	9 (42.9)	21 (25.0)	1.654 (0.726; 3.771)	
Moderate	2 (1.9)	0(0.0)	2 (2.4)	not estimable	

LVDD, left ventricular diastolic diameter; LVSD, left ventricular systolic diameter; SIV, interventricular septum thickness; LVPP, left ventricular posterior wall thickness; LVMI, left ventricular mass index; LVEF, left ventricular ejection fraction; RVDD, right ventricular diastolic diameter; PH, pulmonary hypertension.

Also based on the results of Table 2, a multivariate model was adjusted with the selection, in addition to the variables that presented statistical significance (LVPW and LVMI), those with *p*-value lower than 0.20. Thus, a multivariate model was obtained with the following variables: LV diastolic diameter (LVDD), interventricular septum (IVS) and LVPW thickness, LVMI and the presence of pulmonary hypertension (PH). Based on this adjustment, we found that LVDD, IVS and IMVE were not significant (*p* = 0.858, *p* = 0.853, and *p* = 0.794, respectively), while LVPW and PH were independently associated with rejection (*p* < 0.05). When the LVPW increases 1 mm, the chance of rejection increases 1.685 times (95% CI: 1.185; 2.396 – *p* = 0.004); the chance of rejection among patients with PH is 2.252 times that observed among patients who do not have PH (95% CI: 1.100; 4.607 – *p* = 0.026).

Results obtained by Doppler are shown in Table 3. We observed that the relationship between E and A waves of the categorized mitral flow (E/A) was significantly associated with rejection [OR 2.335 (1.029; 5.296), *p* = 0.042]. We also verified that E/e' was significantly associated with rejection [OR 1.186 (1.118; 1.257), *p* < 0.001].

**Table 3:** Conventional echocardiographic parameters obtained by Doppler assessment (pulse-wave, continuous-wave, color and tissue) according to rejection.

**Echocardiographic parameter**	**General (*n* = 105)**	**Rejection**		**OR (95% CI)**	***p*-value**
		**Present (*n* = 21)**	**Absent (*n* = 84)**		
TR, *n* (%)					0.664
Absent	9 (8.6)	0 (0.0)	9 (10.7)	Not estimable	
Minimal/mild	82 (78.1)	17 (81.0)	65 (77.4)	1,000	
Moderate	14 (13.3)	4 (19.0)	10 (11.9)	1.292 (0.407; 4.097)	
MR, *n* (%)					0.315
Absent	15 (14.3)	1 (4.8)	14 (16.7)	1,000	
Minimal/mild	89 (84.8)	20 (95.2)	69 (82.1)	2.379 (0.439; 12.900)	
Moderate	1 (1.0)	0 (0.0)	1 (1.2)	not estimable	
E (m/s), *n* (%)					0.582
< 0.5	9 (8.6)	1 (4.8)	8 (9.5)	1,000	
≥0.5	96 (91.4)	20 (95.2)	76 (90.5)	1.883 (0.198; 17.933)	
A (m/s)	0.47 ± 0.14	0.50 ± 0.15	0.47 ± 0.14	3.024 (0.201; 45.509)	0.424
E/A	1.71 ± 0.58	1.90 ± 0.62	1.66 ± 0.56	1.466 (0.763; 2.817)	0.251
E/A, *n* (%)					0.042
< 0.8	6 (5.8)	0 (0.0)	6 (7.2)	Not estimable	
0.8–2.0	69 (66.3)	12 (57.1)	57 (68.7)	1,000	
>2.0	29 (27.9)	9 (42.9)	20 (24.1)	2.335 (1.029; 5.296)	
*e'* (m/s), *n* (%)					0.382
< 0.10	48 (52.7)	11 (57.9)	37 (51.4)	1.471 (0.620; 3.489)	
≥0.10	43 (47.3)	8 (42.1)	35 (48.6)	1,000	
E / *e'*	8.4 ± 3.8	10.7 ± 5.1	7.9 ± 3.2	1.186 (1.118; 1.257)	< 0.001

TR, tricuspid regurgitation; MR, mitral regurgitation; E, mitral flow E wave velocity; A, mitral flow A wave velocity; *e'*, mean mitral tissue Doppler *e'* wave velocity; E/*e'*, relationship between the E wave and *e'* velocity.

Quantitative variables obtained by offline 3D analysis regarding rejection are described in Table 4. It was observed that the RV end-diastolic volume (RVEDV) [OR 1.018 (1.007; 1.028), *p* = 0.001], RV stroke volume (RVSV) [OR 1.036 (1.025; 1.048), *p* < 0.001], right ventricular internal diameter, measured at tricuspid level (RVID2) [OR 1.065 (1.008; 1.124), *p* = 0.025], and RV longitudinal diameter (RVID3) [OR 1.088 (1.028; 1.150), *p* = 0.003] were significantly different between patients with and without rejection. However, no multivariate model was possible since those variables are highly correlated with each other.

**Table 4:** Morphological and functional echocardiographic parameters of the right ventricle according to rejection.

**Echocardiographic parameter**	**General (*n* = 105)**	**Rejection**		**OR (95% CI)**	***p*-value**
		**Present (*n* = 21)**	**Absent (*n* = 84)**		
RVEDV (mL)	88.2 ± 23.6	98.0 ± 29.2	85.7 ± 21.6	1.018 (1.007; 1.028)	0.001
RVESV (mL)	51.8 ± 15.8	54.1 ± 15.3	51.2 ± 16.0	1.017 (0.997; 1.037)	0.091
RVSV (mL)	36.3 ± 13.1	43.9 ± 18.4	34.4 ± 10.8	1.036 (1.025; 1.048)	< 0.001
RVEF (%)	41.2 ± 8.5	44.1 ± 7.7	40.5 ± 8.6	1.032 (0.987; 1.079)	0.166
RVID1 (mm)	22.4 ± 4.6	22.4 ± 4.6	22.4 ± 4.6	1.006 (0.919; 1.101)	0.891
RVID2 (mm)	34.4 ± 6.5	36.6 ± 5.2	33.8 ± 6.7	1.065 (1.008; 1.124)	0.025
RVID3 (mm)	71.5 ± 6.3	72.7 ± 8.4	71.1 ± 5.6	1.088 (1.028; 1.150)	0.003
TAPSE (mm)	9.2 ± 4.2	10.2 ± 4.5	8.9 ± 4.2	1.041 (0.955; 1.135)	0.357
FAC (%)	37.0 ± 10.2	40.0 ± 8.0	36.2 ± 10.6	1.012 (0.972; 1.055)	0.551

RVEDV, right ventricular end-diastolic volume; RVESV, right ventricular end-systolic volume; RVSV, right ventricular stroke volume; RVEF, right ventricular ejection fraction; RVID1, right ventricular internal diameter (mid-ventricular); RVID2, right ventricular internal diameter (tricuspid level); RVID3, longitudinal diameter of the right ventricle; TAPSE, tricuspid annulus systolic excursion; FAC, fractional area change of the right ventricle.

RV strain results did not differentiate patients with rejection from those without [OR 1.034 (0.937; 1.143) and OR 1.029 (0.956; 1.109), for septal and free-wall RV strain respectively] (Table 5). Results of 3D LV analysis and LV GLS are described in Table 6. We observed that both GLS and desynchrony index (ID3D) were not associated with rejection [OR 0.984 (0.867; 1.116) and OR 0.950 (0.839; 1.076) respectively]. Three dimensional LVEF was higher in patients with rejection, however it showed only a marginally significant association [OR 1.035 (0.995; 1.077), *p* = 0.085].

**Table 5:** Right ventricular strain, obtained by offline analysis of the three-dimensional full volume according to rejection.

**Right ventricular strain**	**General (*n* = 105)**	**Rejection**		**OR (95% CI)**	***p*-value**
		**Present (*n* = 21)**	**Absent (*n* = 84)**		
RVS-S (%)	10.6 ± 3.8	11.2 ± 3.5	10.5 ± 3.9	1.034 (0.937; 1.143)	0.504
RVS-FW (%)	16.4 ± 5.6	18.5 ± 4.4	15.9 ± 5.8	1.029 (0.956; 1.109)	0.446

RVS-S, right ventricular strain (septum); RVS-FW, right ventricular strain (free wall).

**Table 6:** Strain and three-dimensional assessment of the left ventricle according to rejection.

**Echocardiographic parameter**	**General (*n* = 105)**	**Rejection**		**OR (95% CI)**	***p*-value**
		**Gift (*n* = 21)**	**Absent (*n* = 84)**		
GLS (%)	−14.6 ± 3.2	−15.0 ± 3.3	−14.5 ± 3.2	0.984 (0.867; 1.116)	0.802
LVEF-3D (%)	56.9 ± 10.5	57.6 ± 11.1	56.7 ± 10.4	1.035 (0.995; 1.077)	0.085
ID3D (%)	4.4 ± 3.7	4.0 ± 3.6	4.5 ± 3.7	0.950 (0.839; 1.076)	0.421

GLS, left ventricular global longitudinal strain; LVEF-3D, left ventricular three-dimensional ejection fraction; ID3D, left ventricular three-dimensional desynchrony index.

Regarding the type of graft rejection, we observed a moderate or severe cellular reaction in 9 cases (8.6%) (Table 7). Antibody-mediated (humoral) rejection, diagnosed by immunofluorescence or immunohistochemistry, was found in 13 cases (12.4%) (Table 8).

**Table 7:** Prevalence of cellular rejection to heart graft.

**Cell rejection**	***n* (%)**
Absent	34 (32.4)
Mild cell rejection (1R)	62 (59.0)
Moderate cellular rejection (2R)	9 (8.6)
Severe cell rejection (3R)	0 (0.0)

**Table 8:** Prevalence of antibody-mediated (humoral) rejection to heart graft.

**Humoral rejection (immunofluorescence/immunohistochemistry)**	***n* (%)**
Negative or inconclusive	92 (87.6)
Positive	13 (12.4)

Prevalence of cellular rejection was 8.6% (95% CI: 4%; 15.6%) in our sample, and their echocardiographic parameters and association with cellular rejection are shown in Table 9. Only LVPW and LVMI differentiated exams with cellular rejection from those without [OR 1.825 (1.097; 3.036), *p* = 0.021; and OR 1.033 (1.009; 1.059), *p* = 0.008, respectively]. No Doppler-derived echocardiographic parameter was significantly associated to cellular rejection.

**Table 9:** Conventional echocardiographic parameters obtained by two-dimensional and descriptive evaluation according to cellular rejection.

**Echocardiographic parameter**	**General (*n* = 105)**	**Cell rejection**		**OR (95% CI)**	***p*-value**
		**Present (*n* = 9)**	**Absent (*n* = 96)**		
LVDD (mm)	45.5 ± 3.9	47.2 ± 5.1	45.3 ± 3.8	1.116 (0.978; 1.274)	0.104
LVSD (mm)	28.6 ± 3.0	29.3 ± 3.7	28.5 ± 2.9	1.092 (0.911; 1.310)	0.341
SIV (mm)	10.7 ± 1.6	11.4 ± 0.7	10.7 ± 1.6	1.291 (0.913; 1.826)	0.148
LVPW (mm)	10.2 ± 1.2	11.0 ± 1.0	10.1 ± 1.2	1.825 (1.097; 3.036)	0.021
LVMI (g/m^2^)	96.2 ± 19.8	112.6 ± 23.1	94.7 ± 18.9	1.033 (1.009; 1.059)	0.008
LVEF (%)	66.9 ± 5.4	68.2 ± 3.7	66.8 ± 5.5	1.041 (0.938; 1.156)	0.451
RVDD (mm)	27.5 ± 3.3	27.4 ± 2.3	27.5 ± 3.4	0.986 (0.835; 1.165)	0.871
PH, *n* (%)					0.595
Absent	86 (81.9)	7 (77.8)	79 (82.3)	1,000	
Present (>35 mmHg)	19 (18.1)	2 (22.2)	17 (17.7)	1.404 (0.402; 4.898)	
Pericardial effusion, *n* (%)					0.662
Absent	73 (69.5)	7 (77.8)	66 (68.7)	1,000	
Minimal/mild	30 (28.6)	2 (22.2)	28 (29.2)	0.747 (0.202; 2.763)	
Moderate	2 (1.9)	0 (0.0)	2 (2.1)	not estimable	

LVDD, left ventricular diastolic diameter; LVSD, left ventricular systolic diameter; SIV, interventricular septum thickness; LVPW, left ventricular posterior wall thickness; LVMI, left ventricular mass index; LVEF, left ventricular ejection fraction; RVDD, right ventricular diastolic diameter; HP, pulmonary hypertension.

When it comes to 3D RV parameters, we observed that RVEDV [OR 1.020 (1.005; 1.036), *p* = 0.009], RVSV [OR 1.039 (1.018; 1.060), *p* < 0.001], RVEF [OR 1.067 (1.009; 1.128), *p* = 0.024] and RVID3 [OR 1.082 (1.001; 1.169), *p* = 0.046] significantly differentiated exams with cellular rejection from those without. Results from RV analysis are shown in Table 10. We conducted a multivariate analysis with RVEDV and RVEF. When RVEDV increases 1 mL, chance of cellular rejection increases 1.8% (95% CI: 0.3%; 3.3%; *p* = 0.019) and when RVEF increases 1%, chance of cellular rejection increases 5.4% (95% CI: 0.6%; 10.5%; *p* = 0.027). No associations were observed between RV strain and cellular rejection (Table 11), as well as LV 3D variables and GLS results.

**Table 10:** Morphological and functional echocardiographic parameters of the right ventricle according to cellular rejection.

**Echocardiographic parameter**	**General (*n* = 105)**	**Cell rejection**		**OR (95% CI)**	***p*-value**
		**Present (*n* = 9)**	**Absent (*n* = 96)**		
RVEDV (mL)	88.2 ± 23.6	109.6 ± 26.9	86.1 ± 22.4	1.020 (1.005; 1.036)	0.009
RVESV (mL)	51.8 ± 15.8	59.0 ± 18.1	51.1 ± 15.5	1.019 (0.992; 1.047)	0.173
RVSV (mL)	36.3 ± 13.1	50.6 ± 18.1	35.0 ± 11.8	1.039 (1.018; 1.060)	< 0.001
RVEF (%)	41.2 ± 8.5	45.9 ± 9.8	40.8 ± 8.3	1.067 (1.009; 1.128)	0.024
RVID1 (mm)	22.4 ± 4.6	23.5 ± 4.7	22.3 ± 4.6	1.042 (0.920; 1.180)	0.515
RVID2 (mm)	34.4 ± 6.5	36.7 ± 5.4	34.2 ± 6.5	1.041 (0.954; 1.135)	0.367
RVID3 (mm)	71.5 ± 6.3	75.1 ± 10.0	71.1 ± 5.8	1.082 (1.001; 1.169)	0.046
TAPSE (mm)	9.2 ± 4.2	10.1 ± 5.4	9.1 ± 4.1	1.050 (0.935; 1.178)	0.412
FAC (%)	37.0 ± 10.2	41.1 ± 8.7	36.6 ± 10.3	1.047 (0.993; 1.104)	0.092

RVEDV, right ventricular end-diastolic volume; RVESV, right ventricular end-systolic volume; RVSV, right ventricular stroke volume; RVEF, right ventricular ejection fraction; RVID1, right ventricular internal diameter (mid-ventricular); RVID2, right ventricular internal diameter (tricuspid level); RVID3, longitudinal diameter of the right ventricle; TAPSE, tricuspid annulus systolic excursion; FAC, fractional area change of the right ventricle.

**Table 11:** Right ventricular strain, obtained by offline analysis of the three-dimensional full volume according to cellular rejection.

**Right ventricular strain**	**General (*n* = 105)**	**Cell rejection**		**OR (95% CI)**	***p*-value**
		**Present (*n* = 9)**	**Absent (*n* = 96)**		
RVS-S (%)	10.6 ± 3.8	12.6 ± 3.7	10.4 ± 3.8	1.107 (0.974; 1.258)	0.118
RVS-FW (%)	16.4 ± 5.6	18.1 ± 5.4	16.2 ± 5.6	1.056 (0.967; 1.152)	0.227

RVS-S, right ventricular strain (septum); RVS-FW, right ventricular strain (free wall).

Prevalence of antibody-mediated (humoral) rejection was 12.4% (95% CI: 6.8%; 20.2%) in the studied population. We observed that only LVPW [OR 1.650 (1.028; 2.648), *p* = 0,038] and the presence of PH [OR 3.325 (1.154; 9.583), *p* = 0.026] were significantly associated to humoral rejection. A marginally significant result of association between pericardial effusion and humoral rejection was observed [OR 2.775 (0.996; 7.734), *p* = 0.051]. Other findings are shown in Table 12. A multivariate model was adjusted to assess whether both variables, LVPW and PH, contribute to the explanation of humoral rejection. Based on this adjustment, both variables contributed independently, and when the LVPW increases 1 mm, chance of humoral rejection increases 1.64 times (95% CI: 1.006; 2.675; *p* = 0.047), and chance of humoral rejection among patients with PH is 2.85 times higher than those without (95% CI: 1.013; 8.019; *p* = 0.047).

**Table 12:** Conventional echocardiographic parameters obtained by two-dimensional and descriptive evaluation according to antibody-mediated (humoral) rejection identified by immunofluorescence or immunohistochemistry.

**Echocardiographic parameter**	**General (*n* = 105)**	**Humoral rejection**		**OR (95% CI)**	***p*-value**
		**Present (*n* = 13)**	**Absent (*n* = 92)**		
LVDD (mm)	45.5 ± 3.9	45.1 ± 4.3	45.5 ± 3.9	1.027 (0.882; 1.194)	0.733
LVSD (mm)	28.6 ± 3.0	28.5 ± 2.2	28.6 ± 3.1	0.969 (0.805; 1.168)	0.745
SIV (mm)	10.7 ± 1.6	11.6 ± 1.2	10.6 ± 1.6	1.284 (0.916; 1.802)	0.147
LVPW (mm)	10.2 ± 1.2	11.0 ± 1.0	10.1 ± 1.1	1.650 (1.028; 2.648)	**0.038**
LVMI (g/m^2^)	96.2 ± 19.8	103.6 ± 19.4	95.2 ± 19.8	1.018 (0.993; 1.044)	0.156
LVEF (%)	66.9 ± 5.4	66.5 ± 4.0	66.9 ± 5.6	1.040 (0.951; 1.138)	0.393
RVDD (mm)	27.5 ± 3.3	28.8 ± 2.9	27.3 ± 3.4	1.149 (0.964; 1.289)	0.143
PH, *n* (%)					**0.026**
Absent	86 (81.9)	7 (53.9)	79 (85.9)	1,000	
Present (>35 mmHg)	19 (18.1)	6 (46.1)	13 (14.1)	3.325 (1.154; 9.583)	
Pericardial effusion, *n* (%)					**0.051**
Absent	73 (69.5)	6 (46.1)	67 (72.8)	1,000	
Minimal/mild	30 (28.6)	7 (53.9)	23 (25.0)	2.775 (0.996; 7.734)	
Moderate	2 (1.9)	0 (0.0)	2 (2.2)	Not estimable	

LVDD, left ventricular diastolic diameter; LVSD, left ventricular systolic diameter; SIV, interventricular septum thickness; LVPW, left ventricular posterior wall thickness; LVMI, left ventricular mass index; LVEF, left ventricular ejection fraction; RVDD, right ventricular diastolic diameter; PH, pulmonary hypertension.

When it comes to Doppler-derived echocardiographic parameters and their association with humoral rejection, we observed that E/A [OR 2.044 (1.017; 4.109), *p* = 0.045] and E/*e'* [OR 1.262 (1.153; 1.381), *p* < 0.001] ratios were significantly associated to humoral rejection. A multivariate adjustment was performed and only E/*e'* ratio was associated to humoral rejection (*p* = 0.005).

Regarding 3D variables of the RV, we observed that only RVSV was associated to humoral rejection [OR 1.029 (1.007; 1.052), *p* = 0.011]. We also observed that either RV strain variables (Table 13) or 3D evaluation of LV (Table 14) did not show any significant association to humoral rejection, as well as LV strain values.

**Table 13:** Right ventricular strain, obtained by offline analysis of the three-dimensional full volume according to antibody-mediated (humoral) rejection identified by immunofluorescence or immunohistochemistry.

**Right ventricular strain**	**General (*n* = 105)**	**Humoral rejection**		**OR (95% CI)**	***p*-value**
		**Present (*n* = 13)**	**Absent (*n* = 92)**		
RVS-S (%)	10.6 ± 3.8	10.5 ± 3.1	10.6 ± 4.0	0.977 (0.857; 1.113)	0.723
RVS-FW (%)	16.4 ± 5.6	18.6 ± 3.7	16.1 ± 5.8	1.028 (0.937; 1.128)	0.558

RVS-S, right ventricular strain (septum); RVS-FW, right ventricular strain (free wall).

**Table 14:** Strain and three-dimensional assessment of the left ventricle according to antibody-mediated (humoral) rejection identified by immunofluorescence or immunohistochemistry.

**Echocardiographic parameter**	**General (*n* = 105)**	**Humoral rejection**		**OR (95%CI)**	***p*-value**
		**Present (*n* = 13)**	**Absent (*n* = 92)**		
GLS (%)	−14.6 ± 3.2	−14.1 ± 3.1	−14.7 ± 3.3	1.061 (0.905; 1.244)	0.466
LVEF-3D (%)	56.9 ± 10.5	54.3 ± 8.0	57.3 ± 10.8	0.994 (0.949; 1.041)	0.802
ID3D (%)	4.4 ± 3.7	4.3 ± 3.6	4.4 ± 3.7	0.950 (0.817; 1.105)	0.509

GLS, left ventricular global longitudinal strain; LVEF-3D, three-dimensional left ventricular ejection fraction; ID3D, three-dimensional left ventricular desynchrony index.

## Discussion

The results of our research showed no association between RV and LV myocardial deformation and graft rejection in heart transplant patients. Although the observations found are different from the initial expectation regarding the importance of right ventricular strain in cases of cardiac graft rejection, the present study was a pioneer in the classification of the type of rejection after HT and its correlation with the echocardiographic findings derived from the ST. Most of the studies found in literature that deal with rejection after HT use cellular type as an outcome. Studies with advanced echocardiography in post-HT patients have also been published, although they do not specify which type of rejection is more prevalent. Our series reveals, based on the differentiation of the type of rejection, a higher prevalence of antibody-mediated rejection. To date, we are not aware of other studies that address this rejection classification, which highlights the relevance and originality of our research.

According to the findings of Cruz et al. (19), the myocardial deformation of both ventricles is reduced (in absolute values) in patients submitted to HT who presented with rejection. Unlike that research, where LV deformation was composed by three different types of strain (longitudinal, radial, and circumferential) and RV strain revealed deformation data only from the free wall, our study used the right ventricular strain derived from the three-dimensional image, which resulted in a composed strain data, split into free wall and septum strain. In addition, it is known that there are differences between the results obtained by software from different vendors, which may have influenced the failure of the reproducibility of those results. Furthermore, our research aimed to identify, if present, association of different echocardiographic parameters and the two types of rejection, while the study from Cruz et al. was limited to the cellular one.

A study with heart transplant patients published by Marciniak et al. (24), using TDI-derived strain analysis, observed a reduction in the absolute value of RV free wall strain in patients with cellular rejection, which was not reproduced in our population. This can be explained by the difference in physical properties between the image acquisition methods, with ST more accurate because it is not influenced by the image acquisition angle. Eleid et al. (12) observed, in another study that carried out a 2-year follow-up of transplanted patients, a reduction in all absolute values of strain derived from the TDI, regardless of the presence of rejection. Such heterogeneity of results may be associated with changes in thoracic anatomy inherent to the surgical procedure.

The present work, in its analysis of the left ventricular GLS, found no evidence of association between the results obtained with this technology and the presence of graft rejection, whether cellular or humoral. Ambardekar et al. (25) failed to correlate graft rejection with GLS in patients after 1 year of HT, and suggested that this method could not replace myocardial biopsy for the diagnosis of graft rejection, and that new non-invasive methods for this diagnosis are needed. Despite our study comprising a more robust series, having a prospective design, and adding three-dimensional parameters of the LV and right ventricular strain, we obtained similar results when trying to correlate these data with rejection. This further highlights the need for continuity in studies that apply this technology to heterogeneous populations such as heart transplant recipients.

The role played by GLS in HT patients who develops graft rejection can be amplified by adding circumferential deformation in the equation. Global circumferential strain (GCS) of those patients who develops antibody-mediated rejection, according to Ciarka et al. (26), show a progressive decrease up to 6 months before clinically apparent rejection. A similar behavior of LV function, in their study, is seen in GLS findings. Those reduced strain values could diagnose a subclinical stage of rejection and allow an early optimization of immunosuppression, preventing progression of the graft rejection. However, we could not identify any association between lower GLS and rejection of any type in our study. Our population showed no grade 3 cellular rejection, compared to exclusive severe rejection individuals who were included in their sample. Despite the growing relevance of GCS in LV function, that parameter was not studied, once GCS is not available in most echocardiography labs in our country.

In our study, we identified the association between RVEDV (*p* = 0.001) and RVSV (*p* < 0.001), derived from three-dimensional analysis, with graft rejection, demonstrating the relevance of right ventricular assessment in the identification of cellular rejection. D'Andrea et al. (27) had already suggested a high accuracy of the geometric assessment of the RV using the three-dimensional method, compared to magnetic resonance imaging, for functional assessment of the graft after HT. It is important to point out that this three-dimensional evaluation overcomes the limitations of other techniques of functional analysis of the RV, such as the TDI, in which the dependence of the acquisition angle interferes with the accuracy of the measurements. In a scenario where the patient profile, in most cases, presents with technical difficulties in obtaining aligned images for evaluation, the independence of the acquisition angle represents a greater possibility of accuracy.

Still regarding the RV volumes obtained through the three-dimensional full volume, we identified in our results that the increase in diastolic volumes and RVEF are associated with rejection, which may suggest a paradox. The parameterization of the systolic function derived from volumes and their relationships with each other can lead to the false impression that the numerical increase is equivalent to the best function; however, it is common sense that RV measurements may be increased after HT, without this finding indicating graft dysfunction.

The use of three-dimensional reconstructions of the LV has been part of the diagnostic arsenal for estimating cardiac function in the most diverse populations, which includes HT patients. In our results, we found that ID3D did not show association with rejection, maintaining values close to normal in all sub analyses. Chinali et al. (28) in their study on ventricular mechanics in transplant patients, using the 16-segment ID3D, observed a global reduction in ventricular synchrony among transplanted patients, regardless of the presence of rejection. On the other hand, Pan et al. (21) in their research based in rejection and ventricular synchrony, observed that ID3D can be a marker of the clinical deterioration that accompanies graft rejection. It is possible that tachycardia, common in HT patients, have some influence on that difference found in the literature. Assessing the electromechanical coupling of HT patients remains a challenge.

Assessment of conventional echocardiographic parameters in our series showed a relevant association of LVPW thickness (*p* = 0.005) and LVMI (*p* = 0.001) with the presence of rejection of any type. Splitting rejection events into subtypes, we observed that these same parameters still have statistical relevance in the association with cellular reaction (*p* = 0.021 and *p* = 0.008, respectively). In the specific evaluation of humoral rejection results, an association was observed with posterior wall thickness (*p* = 0.038) and with the presence of HP (*p* = 0.026), with a borderline significant *p*-value for pericardial effusion. In 2009, Estep et al. (29), in a multimodality assessment of the HT grafts, had already observed a higher prevalence of increased myocardial thickness in patients with rejection. In their research, they also identified that patients with cellular rejection present pericardial effusion in a greater frequency. Although pericardial effusion is a frequent variable among patients with rejection in the study by Sun et al. (30), that finding was not verified in our sample of positive EMBs for cellular reaction. In another study, Roshanali et al. (31) did observed an association between myocardial thickness and cellular rejection, and also purposed an echocardiographic rejection index, with left ventricular strain parameters as independent factors. A retrospective observation recently published by Bacal et al. (32) who evaluated patients from the HT program at the Albert Einstein Hospital also identified the association of increased myocardial thickness with rejection. In addition to those parameters, obtained from TTE, the authors identified that biochemical alterations, such as C-reactive protein, play a role as a marker of rejection. Despite that significant finding in a highly similar population, biochemical lab results were not present in the initial scope of our research.

Still in the list of conventional echocardiographic variables, we could observe a significant correlation with the presence of graft rejection between the ratio of E and A velocities of the mitral flow, and between the E velocity and the *e'* wave of the TDI (*p* = 0.042 and *p* < 0.001, respectively), which denotes some damage to the diastolic function of those patients with positive biopsies. These findings corroborate what Mena et al. (33) considered relevant in their systematic review on diastolic dysfunction in HT patients. Palka et al. (34), in 2005, had already identified that changes in diastole, measured by the TDI, through the E/*e'* ratio, would be associated with the development of acute rejection. The evaluation of the results of positive biopsies for humoral reaction also showed this correlation; however, when we specifically evaluated cell-type rejection, this association was not observed. This divergence in the results may be related to the higher prevalence of humoral rejection in population of ours.

The final population included in the study was smaller than the number of biopsies performed at the institution within the same period, as 18 exams (~15% of the sample) that had inadequate echocardiographic view for acquiring satisfactory images for analysis were excluded. As described by Bacal et al. (35), that finding is not uncommon, once the disproportion between the size of the graft and the recipient's mediastinum is highly frequent. When that mismatch occurs, the final position of the heart in the chest makes it difficult to record high-quality images. The same difficulty can be present when performing a bedside TTE, where poor quality images are linked to the limited mobilization, mainly when HT patients are in intensive care units or have been just submitted to EMB and still with limited movements.

Recently, Carrion et al. (18) evaluated 19 HT patients from other Brazilian reference center. In that study, 257 EMBs were performed and 87 (~34% of the sample) met exclusion criteria. The number of EMBs performed by each patient was evidently higher than in our series. This fact may be related to the lack of uniformity in the follow-up protocols among HT centers. Orrego et al. (36), in 2012, compared the performance of EMBs periodically up to 2 years after surgery with the shortening of that periodic invasive follow-up to 6 months, regardless of the presence of rejection, and they did not identify any impairment in identification of complications.

Most studies in the literature with a HT population present inference related to general rejection or just the cellular type. Despite this, in 2004, Subherwal et al. (4) claimed attention to a type of non-cellular rejection of HT. Initially established by clinical criteria, antibody-mediated rejection proved to be important in the follow-up of those patients. Our research sought to analyze this type of rejection separately, intended to identify any association with echocardiographic findings, given the higher prevalence of humoral rejection in our series.

In the present study, cellular rejection followed the characterization defined by the International Society of Heart and Lung Transplantation (ISHLT) (5). Graded at 0, 1R, 2R, and 3R, according to the intensity of the inflammatory process identified in EMB, the mildest cellular rejection (1R) does not cause clinical changes or determine alterations in medication management. In many studies, such as the one by Mingo-Santos et al. in 2015 (37), the 1R classification was considered as non-rejection. In their study, evaluating HT patients for the presence of cellular rejection, the prevalence of EMBs 2R or greater was only 5.1%. In comparison, our results, following the same classification criteria, found 8.6% of positive biopsies and, therefore, clinically significant. On the other hand, if we consider any degree of rejection as a positive outcome, rejection in the Mingo-Santos analysis is present in 26.4% of the sample, compared to 67.6% of 1R or 2R graded results in our study. Despite this higher prevalence, the present study did not observe severe rejection (3R) among transplant patients who underwent BEM from December 2017 to January 2020.

Another data capable of impacting the results of the HT is the time of cold ischemia of the graft, directly related to the logistics described and documented in the 3rd. Brazilian Guideline on Heart Transplantation (35). Pickering et al. (38), in 1990, had already described deleterious effects of more than 4 h of cardioplegia, with the development of fibrosis between the myocytes, and related to a higher incidence of rejection. Unfortunately, in our study, this data was not present in most medical records that contained a description of the procedure.

Regarding the association of antibody-mediated (humoral) rejection, recent developments in diagnostic quantification methods cannot yet be translated into the volume of publications. In 2009, Tan et al. (39) described the association of immune complex deposits and the diagnosis of humoral rejection. EMB specimens evaluated by immunofluorescence through complement activation (C4d and C3d), when present, were associated with the onset of ventricular dysfunction. According to Berry et al. (7), immunofluorescence reactivity can be quantified as low reactive (<10% of the specimen), moderately reactive (between 10 and 50% of the specimen) and positive (>50% of the specimen). In our not very robust sample, we chose to consider any reactivity > 10% as positive. In this scenario, we observed positivity for humoral rejection in 12.4% of the cases. Literature data show prevalence of humoral rejection ranging from 3 to 85%, and this statistical range may be related to the absence of established consensus for humoral rejection (40).

We consider limitations of the present study the fact that it conducted in a single HT reference center, with a non-uniform operative technique. Data that could influence the appearance of rejection, such as ischemia time and previous heart disease and comorbidities, were not studied. The small number of rejection cases within our sample can also be considered a limiting factor to the reproducibility of the present study. The presence of laboratory results, such as measurement of C-reactive protein, troponin and atrial natriuretic peptide, concomitant with histopathological findings, could add scientific value to the found observations. Certainly, more studies related to the echocardiographic evaluation of heart transplant patients should be conducted, in order to scientifically support the investment in a non-invasive method of early identification of rejection. With the promising results of the technology currently available, it is possible to reduce the burden of EMB, both in terms of the risks of the procedure itself and the resources provided for its performance, aiming primarily at the benefit of the heart transplant patient.

## Conclusion

The authors concluded that some conventional echocardiographic parameters, such as LVPW thickness and LV mass index, appears to be significantly associated to rejection after heart transplantation. In addition, assessment of diastolic function has an important role in rejection, as shown by E/A and E/e' ratios. When it comes to antibody-mediated type of rejection, LVPW thickness and presence of pulmonary hypertension were significantly related to positive results after endomyocardial biopsy. We did not observe strong association of advanced echocardiographic parameters, as longitudinal strain from both ventricles or 3D variables, and rejection of any type in our sample.

## Data availability statement

The raw data supporting the conclusions of this article will be made available by the authors, without undue reservation.

## Ethics statement

The studies involving human participants were reviewed and approved by Comitê de Ética em Pesquisa do Hospital Israelita Albert Einstein. The patients/participants provided their written informed consent to participate in this study.

## Author contributions

RC, SM, AR, CM, FB, and MV contributed for the concept and design of the study. RC, CF, EF, and AR collected and organized the database. AC performed endomyocardial biopsies. RC and CF were responsible for data analysis and drafting. SM, AR, and MV reviewed manuscript sections. All authors read and approved the submitted version.

## Conflict of interest

The authors declare that the research was conducted in the absence of any commercial or financial relationships that could be construed as a potential conflict of interest.

## Publisher's note

All claims expressed in this article are solely those of the authors and do not necessarily represent those of their affiliated organizations, or those of the publisher, the editors and the reviewers. Any product that may be evaluated in this article, or claim that may be made by its manufacturer, is not guaranteed or endorsed by the publisher.
